# A new classification of personal protective equipment in healthcare settings: Enhancing infection control and prevention

**DOI:** 10.1016/j.infpip.2025.100460

**Published:** 2025-03-29

**Authors:** Hui Jin, Qun Lu, Kaiwen Ni, Xiaoping Ni

**Affiliations:** a Department of Disinfection Surveillance and Vector Control, Hangzhou Centre for Disease Control and Prevention (Hangzhou Health Supervision Institution), Hangzhou, Zhejiang, 310021, China; b Department of Infection Control, Second Affiliated Hospital of Zhejiang University School of Medicine, Hangzhou, Zhejiang, 310009, China

*Dear Editor*,

On 18 April 2024, the World Health Organization (WHO) officially released the *Global Technical Consultation Report on Proposed Terminology for Pathogens that Transmit Through the Air* on its website [1]. This report highlights three critical points. First, infectious respiratory particles (IRPs) are dispersed through exhaled air when an infected individual breathes, speaks, sings, spits, coughs, or sneezes, entering the ambient air. Second, once released, IRPs can potentially infect others via three primary transmission routes: airborne/inhalation, direct deposition, and contact transmission. Finally, the updated terminology shifts away from categorising IRPs strictly by size, instead recognising particle size as a continuum. These advancements will notably influence technical fields, particularly infection prevention and control in healthcare settings, aiming to curb the spread of pathogenic microorganisms.

Amidst the COVID-19 pandemic, regions like China [2], the United States [3], and Europe [4] issued technical guidelines for selecting and using personal protective equipment (PPE) in healthcare and public settings. With the release of the WHO report, it is anticipated that nations and academic institutions will update their PPE selection and usage guidelines to align with the new understanding of respiratory disease transmission via IRPs.

When choosing and wearing PPE, it is essential to understand not just the transmission routes but also the specific roles of different PPE in preventing infections or contamination. This clarity empowers both healthcare professionals and the general populace to make informed PPE choices during infectious disease outbreaks, thereby minimizing infection risks while avoiding over-protection.

We propose a novel classification method for PPE, divided into two primary categories: preventing infection equipment (PIE) and preventing contamination equipment (PCE). PIE is designed to block infectious agents from entering the human body. Its failure or misuse can elevate infection risk, often with limited remedial options; for example, there is a lack of effective vaccines and prophylactic drugs to deal with severe acute respiratory syndrome coronavirus 2 (SARS-CoV-2) infection. Conversely, PCE is meant to protect the human body surface from pathogen contamination. Its malfunction or improper use does not increase infection risk, as effective decontamination methods can mitigate any potential harm; for instance, pathogens can be removed by practicing good hand hygiene, changing clothes, or showering. When PPE is visibly heavily contaminated or has been exposed to multiple patients, appropriate decontamination measures must be implemented, and PCE should be used correctly to ensure maximum protective effectiveness.

Using this framework, PPE can be categorised based on their specific roles in combating infectious agents. For instance, in preventing SARS-CoV-2 transmission, face masks are categorized as PIE, while isolation gowns function as PCE (Table I). However, in the context of preventing HIV transmission through sharp injuries, gloves are classified as PIE. This illustrates how the functionality of PPE components can vary depending on the transmission route.

**Table I:** Role of various PPEs in preventing the spread of SARS-CoV-2

Serial number	Names of PPE components	Function
1	Face mask	PIE
2	Respirator	PIE
3	Goggles	PIE
4	Face shield	PCE/PIE
5	Gloves	PCE
6	Gown	PCE
7	Overall	PCE
8	Apron	PCE
9	Hair cover	PCE
10	Shoe cover	PCE
11	Head cover	PIE/PCE
12	Powered air-purifying respirator	PIE/PCE

PPE, personal protective equipment; PIE, preventing infection equipment; PCE, preventing contamination equipment; SARS-CoV-2, severe acute respiratory syndrome coronavirus 2.

As shown in Table I, certain PPE serves dual purposes. Face shields, for example, not only prevent IRPs from directly depositing on exposed facial mucosa (mouth and nose), functioning as PIE, but also protect the facial skin from contamination, acting as PCE.

This new classification system offers a more transparent understanding of each PPE component's role in infection and contamination prevention, guiding medical personnel and the public to make informed PPE choices based on specific disease transmission routes.

## Ethics

Not required.

## Funding sources

This research was supported by the Basic Public Welfare Research Program of Zhejiang Province (grant number LGF21H260008), Hangzhou Science and Technology Plan Guidance Project (Agriculture and Society) (grant number 20211231Y080), Hangzhou Science and Technology Plan Guidance Project (Agriculture and Society) (grant number 20201231Y058).

## Conflict of interest statement

The authors declare no conflict of interest.
